# Internal Herniation Through the Transverse Mesocolon Causing Small Bowel Obstruction: A Case Report

**DOI:** 10.7759/cureus.89013

**Published:** 2025-07-29

**Authors:** Husham Bakry, Prabu Shankar Sivagnanam, Omar Hamad

**Affiliations:** 1 General Surgery, King Hamad University Hospital, Bahrain Defence Force Royal Medical Services, Busaiteen, BHR; 2 Medicine and Surgery, Royal College of Surgeons in Ireland - Medical University of Bahrain, Busaiteen, BHR

**Keywords:** breast cancer, internal hernia, mesenteric defect, peritoneal metastasis, small bowel obstruction, transmesocolic hernia

## Abstract

Internal hernias are one of the rare causes of small bowel obstruction. Among them, transmesocolic hernias are particularly uncommon and pose a diagnostic challenge due to their non-specific clinical and radiological features. We report the case of a 68-year-old female with known metastatic invasive lobular carcinoma of the breast, who presented with right flank pain, nausea, and constipation. Imaging suggested small bowel obstruction without a clear etiology. Emergency laparotomy revealed a transmesocolic hernia with two jejunal loops herniated through a 6 cm × 3 cm mesocolic defect. The herniated bowel was viable and reduced, and the defect was repaired. No bowel resection was necessary. Transmesocolic hernias may be congenital or acquired. In this case, the defect was likely secondary to peritoneal carcinomatosis or mass effect from metastatic disease. Diagnosis is often delayed due to non-specific signs. Early surgical intervention is essential to prevent bowel ischemia and infarction. Transmesocolic hernia, though rare, should be considered in patients with small bowel obstruction and no prior surgical history, especially in the setting of peritoneal malignancy. High clinical suspicion and timely operative management are critical for favorable outcomes.

## Introduction

An internal abdominal hernia is defined as the protrusion of a viscus through a normal or abnormal peritoneal aperture within the abdominal cavity [1,2]. Despite their rarity, accounting for <1% of all hernias, internal hernias can have a high mortality rate (up to 50%) if complications such as strangulation or bowel infarction occur [3].

Mesenteric defects can be congenital or acquired. According to Meyer’s classification, internal hernias are categorized based on location: paraduodenal (53%), pericaecal (13%), foramen of Winslow (8%), transmesenteric and transmesocolic (8%), intersigmoid (6%), and retroanastomotic (5%) [4]. Among transmesenteric hernias, transmesocolic is the most common subtype. Due to their non-specific clinical and imaging findings, diagnosis requires a high index of suspicion. Early surgical intervention is critical and can significantly influence outcomes.

We present a case of a transmesocolic hernia in a patient with metastatic lobular breast carcinoma, presenting with small bowel obstruction and no significant history of trauma or prior abdominal surgery.

## Case presentation

A 68-year-old female, a known case of invasive lobular carcinoma of the left breast with known metastases to the peritoneum and ovaries, on hormonal therapy, presented to the emergency department with complaints of right flank pain for 24 hours associated with constipation and nausea for two days, with no vomiting, dysuria, or abdominal trauma at presentation. She had a past surgical history of cystoscopy with right ureteric stenting for right ureteric extrinsic compression by metastatic pelvic mass three months prior to her presentation. On physical examination, she was hemodynamically stable with no signs of dehydration. Her abdomen was soft, lax, non-distended, with no tenderness or rigidity. Her blood investigations showed normal total leukocyte count and C-reactive protein, normal renal and liver function tests, with mild hyponatremia and hypoalbuminemia (Table 1).

**Table 1:** Laboratory results with reference ranges

Parameter (Unit)	Patient value	Reference range
Total leucocyte count (x 10^9/L)	5.05	4-11
Absolute neutrophil count (x 10^9/L)	3.71	1.5-8
C-reactive protein (mg/dl)	7.7	0-3
Urea (mmol/L)	1.2	3-7
Creatinine (µmol/L)	52.69	49-90
Sodium (mmol/L)	134	137-148
Potassium (mmol/L)	4.2	3.5-5.1
Magnesium (mmol/L)	0.72	0.7-1
Albumin (g/L)	29.6	38-50
Total bilirubin (µmol/L)	5.5	-
Direct bilirubin (µmol/L)	2.07	0-5
Alkaline phosphatase (U/L)	85	30-130
Alanine aminotransferase (U/L)	30	10-63
Aspartate aminotransferase (U/L)	21.5	10-34
Gamma glutamyltransferase (U/L)	17.2	5-55
Lactate (arterial blood) (mmol/L)	1.6	0.6-1.4

**Figure 1:**
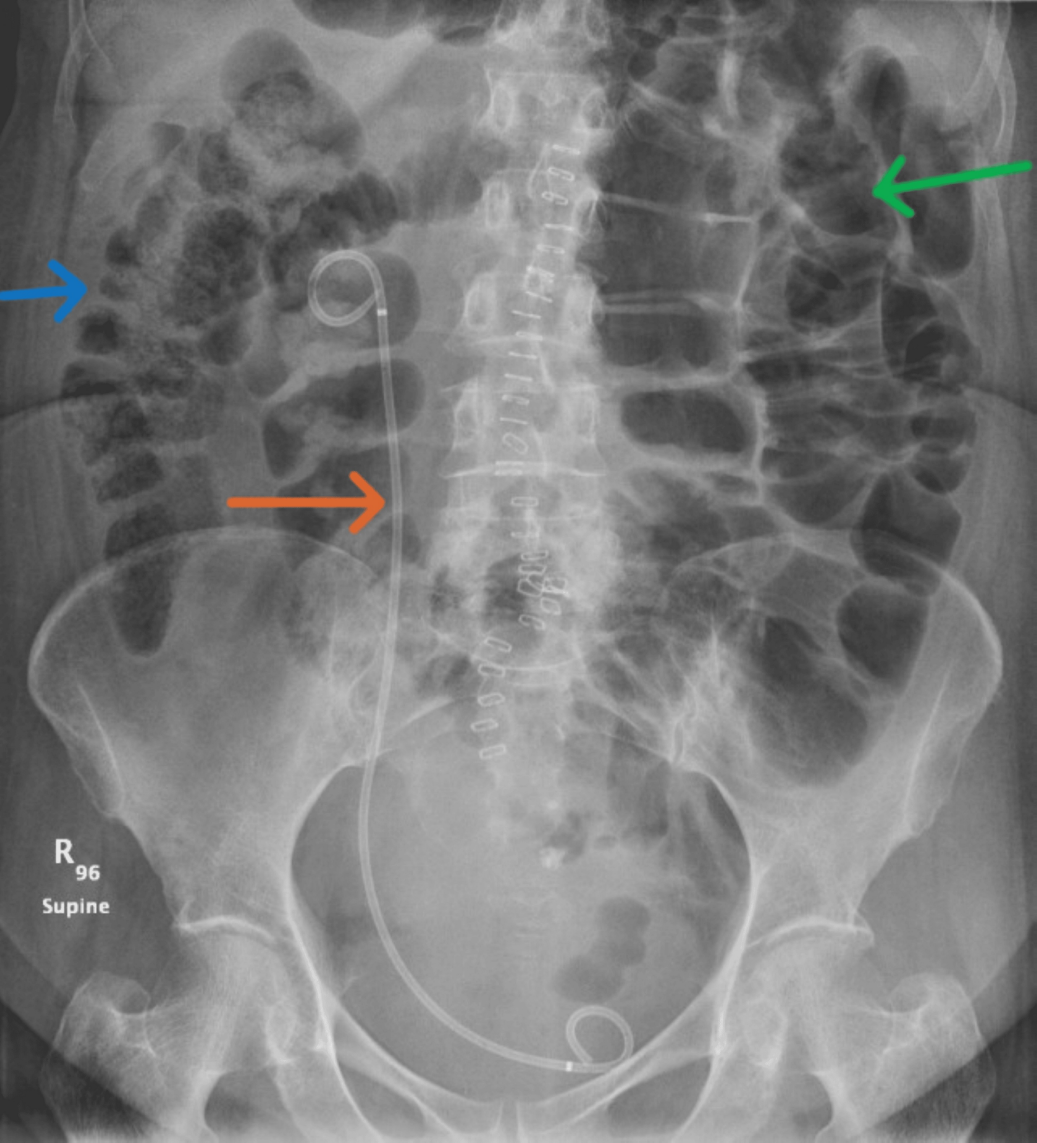
Supine abdominal X-ray The image demonstrates a fecal-loaded colon (blue arrow), dilated small bowel loops (green arrow), and right ureteric stent (orange arrow).

**Figure 2:**
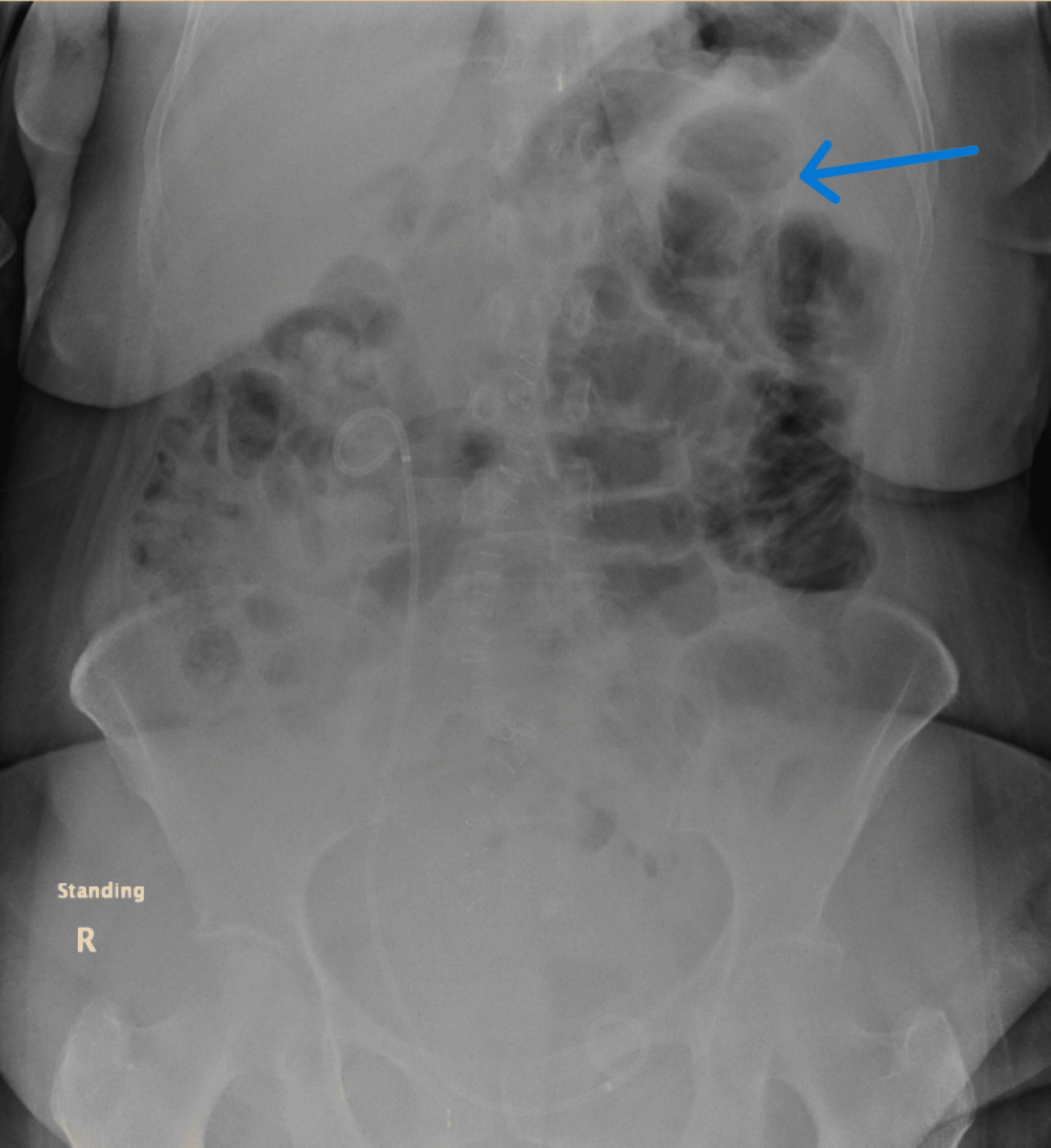
Erect abdominal X-ray The image demonstrates dilated small bowel loops in the left upper quadrant (blue arrow).

An erect chest X-ray showed no pneumoperitoneum. Supine abdominal X-ray (Figure 1) showed a fecal-loaded colon with jejunal dilatation, and the erect abdominal X-ray (Figure 2) showed no air-fluid levels.

Bedside abdominal ultrasound showed mild right renal pelvicalyceal fullness and minimal pelvic ascites.

The patient was initially managed conservatively with nil per oral, intravenous fluids, and symptomatic management. Twelve hours post-admission, the patient developed persistent vomiting (20 episodes) with colicky abdominal pain. Nasogastric decompression drained 250 mL of bilious content.

**Figure 3:**
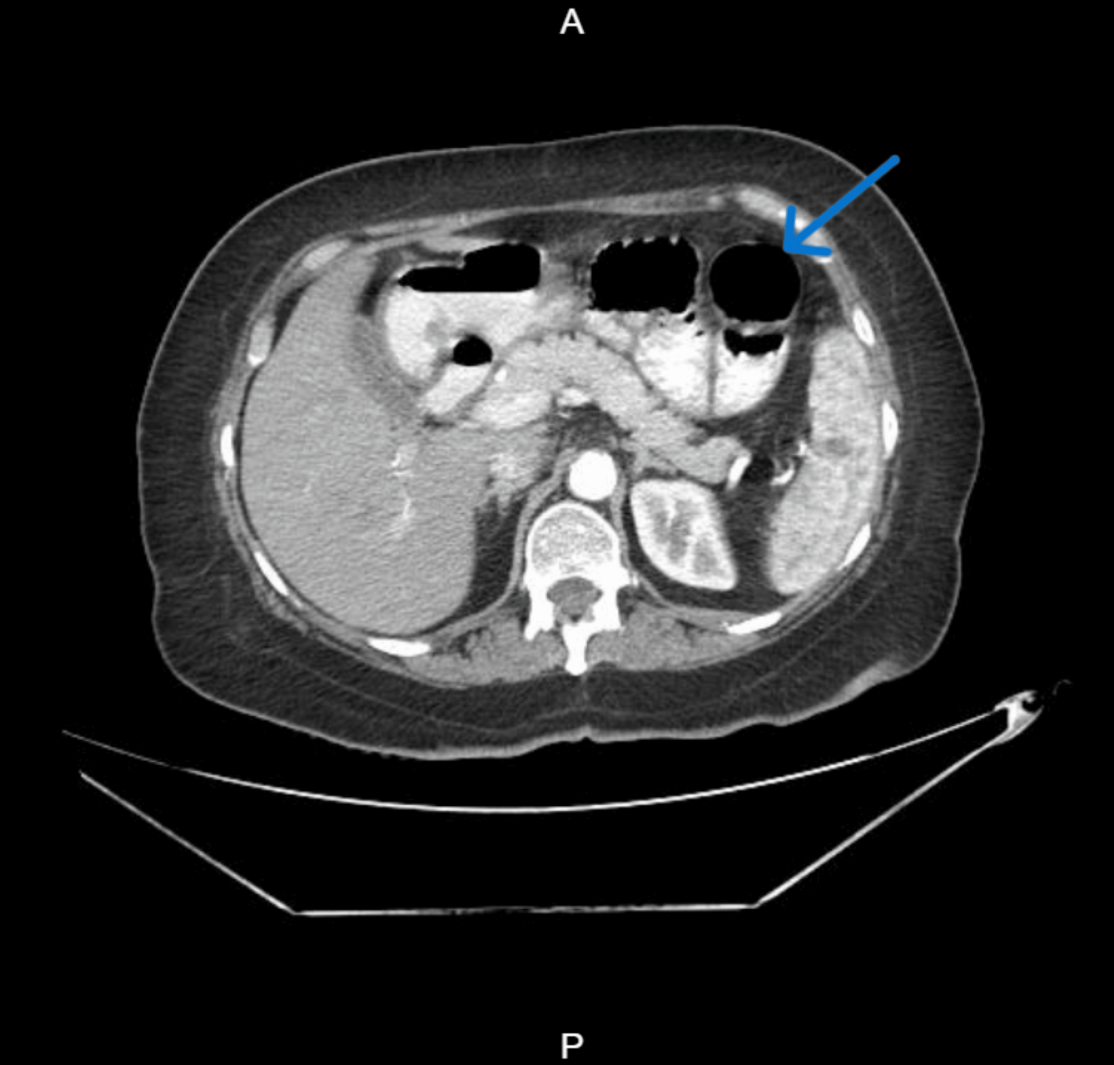
Contrast-enhanced computed tomography (CECT) of the abdomen and pelvis, axial view Dilated jejunal loops (blue arrow) in the lesser sac.

**Figure 4:**
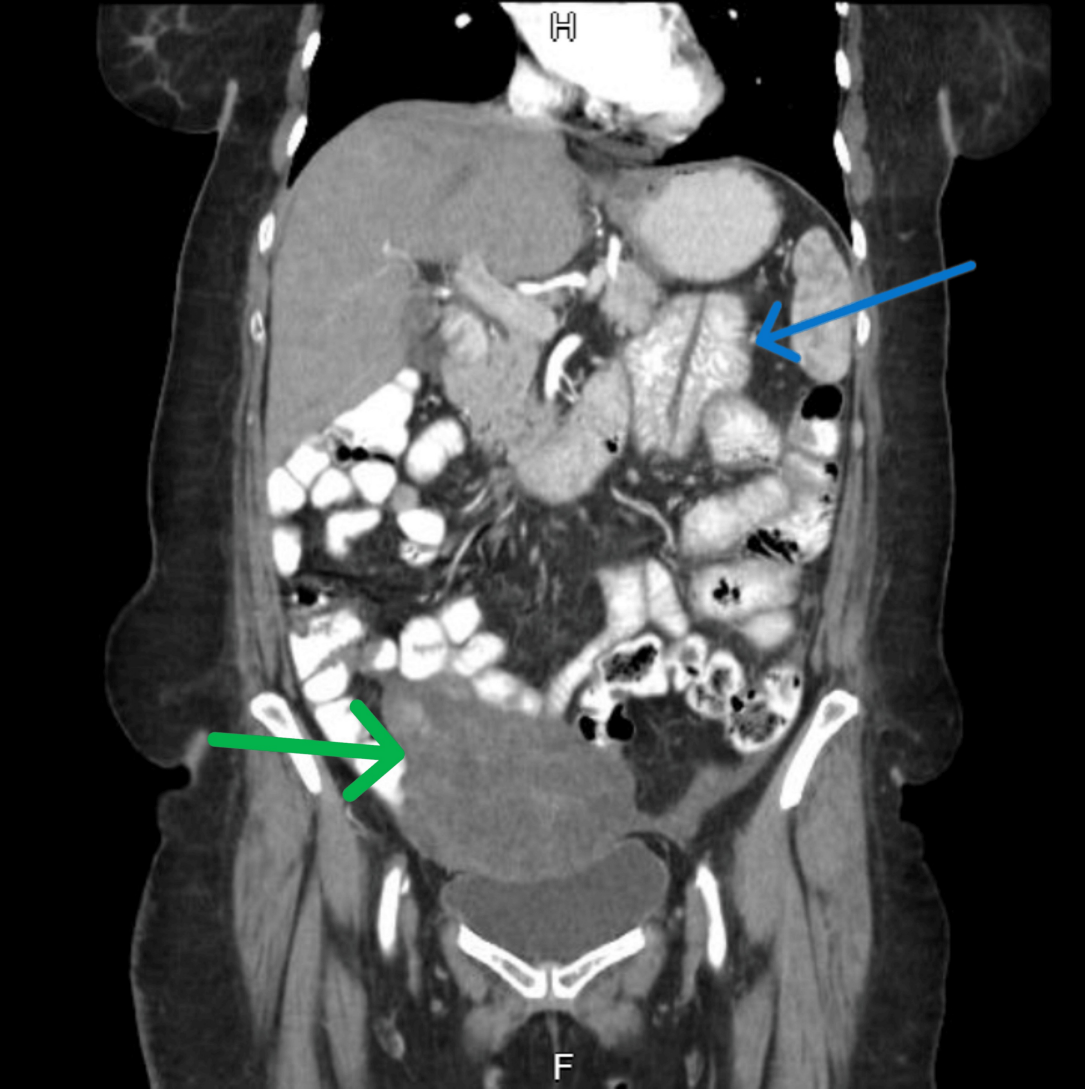
Contrast-enhanced computed tomography (CECT) of the abdomen and pelvis, coronal view Dilated jejunal loops (blue arrow) in the lesser sac and metastatic pelvic deposit (green arrow).

**Figure 5:**
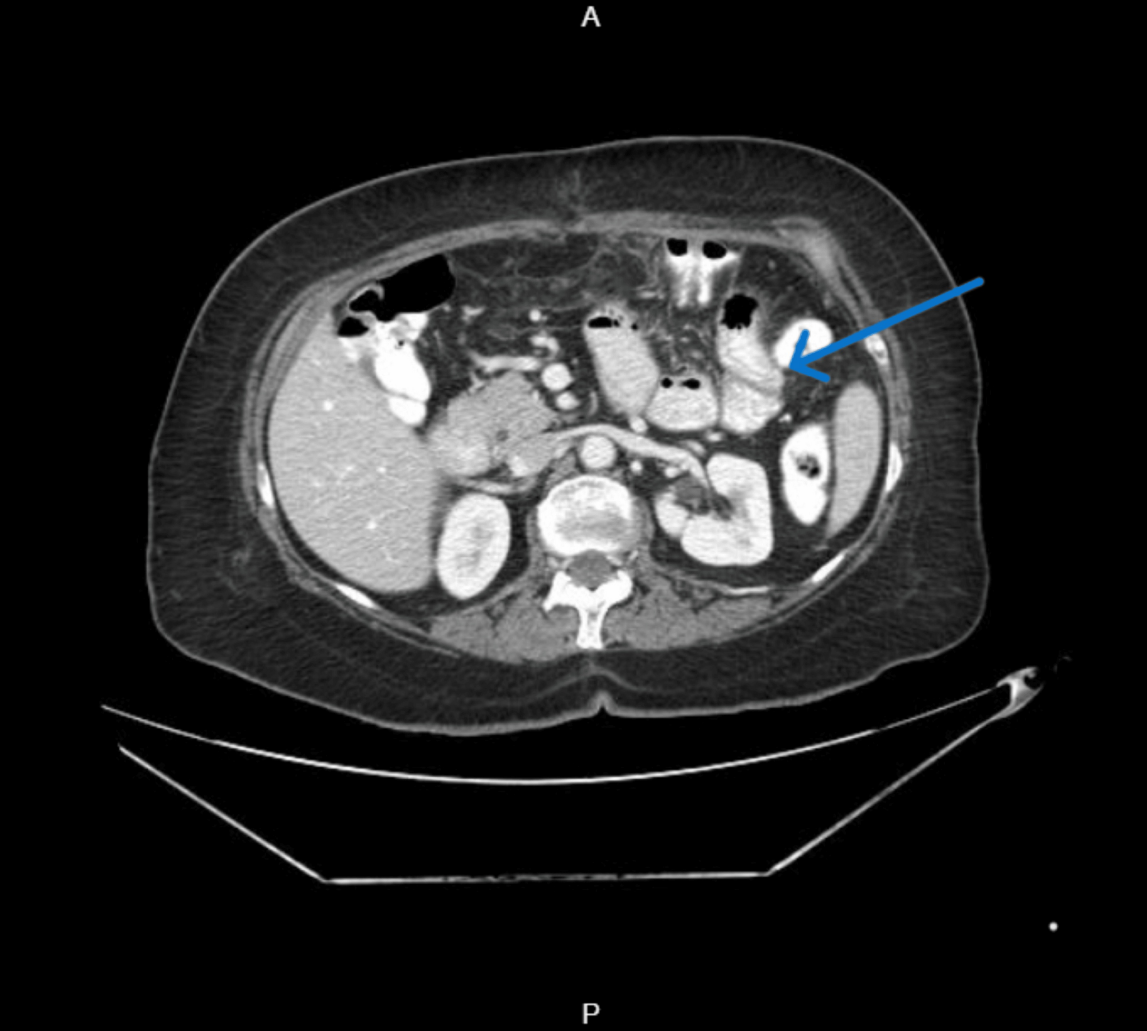
Contrast-enhanced computed tomography (CECT) of the abdomen and pelvis, axial view Transition point (blue arrow) in the jejuno-ileal junction

Contrast-enhanced computed tomography (CECT) of the abdomen and pelvis (Figures 3, 4) revealed dilated proximal jejunal loops up to 4 cm with fecal signs in the small bowel and transition point (Figure 5) at the jejuno-ileal junction, suggestive of closed-loop small bowel obstruction.

**Figure 6:**
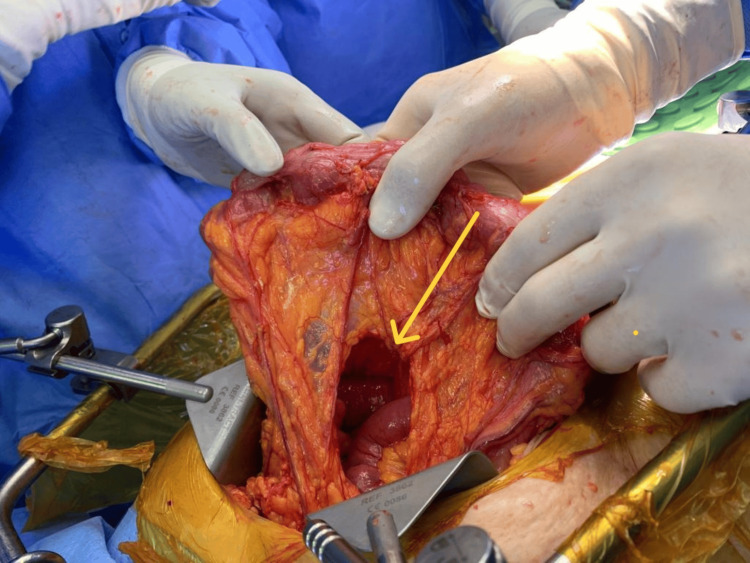
Transmesocolic defect Intraoperative image showing a transmesocolic defect (yellow arrow) of size 6 cm x 3 cm.

**Figure 7:**
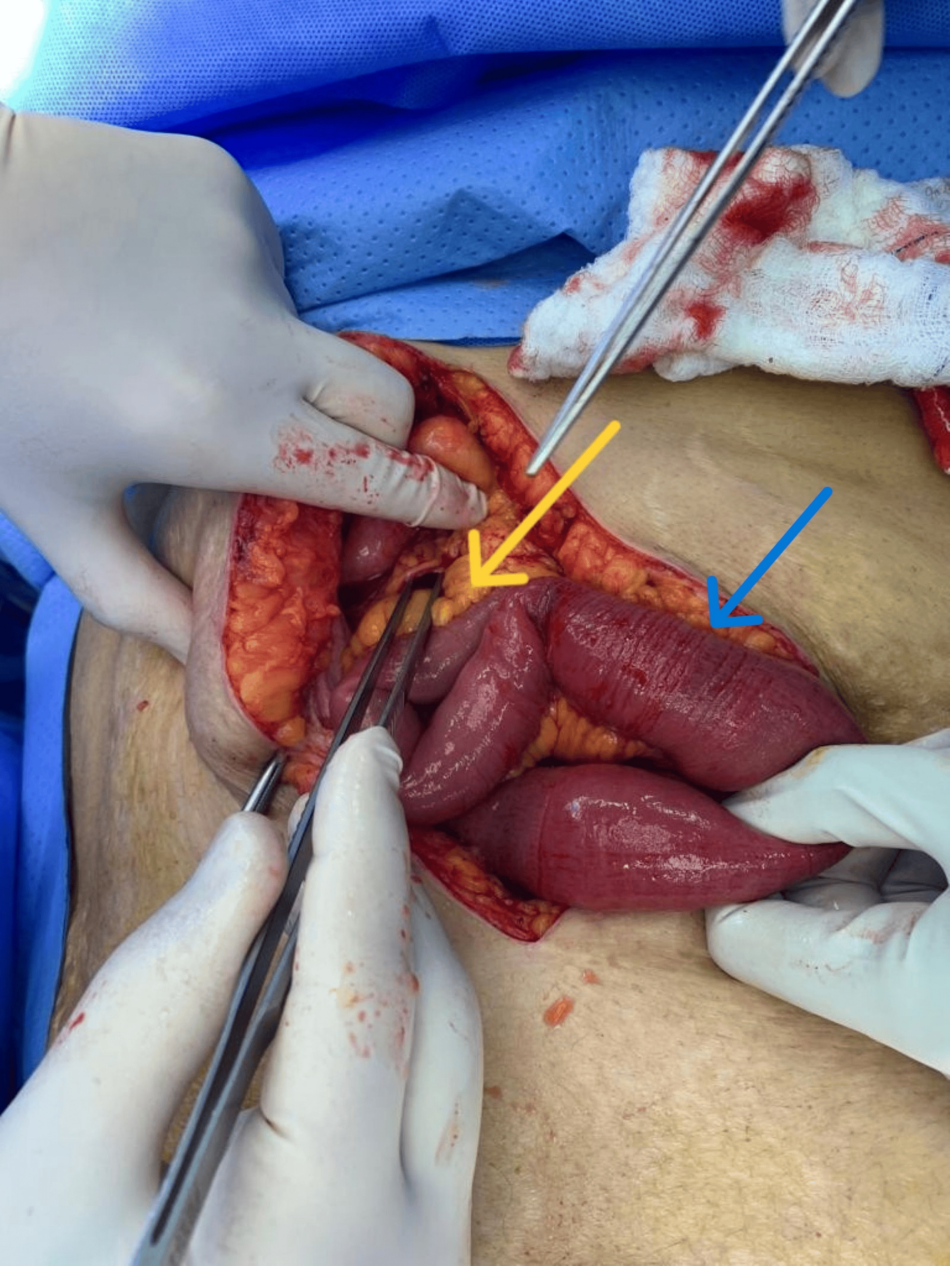
Herniated bowel loop Intraoperative image showing herniated bowel loops (blue arrow) through the transmesocolic defect (yellow arrow) appeared congested but viable.

**Figure 8:**
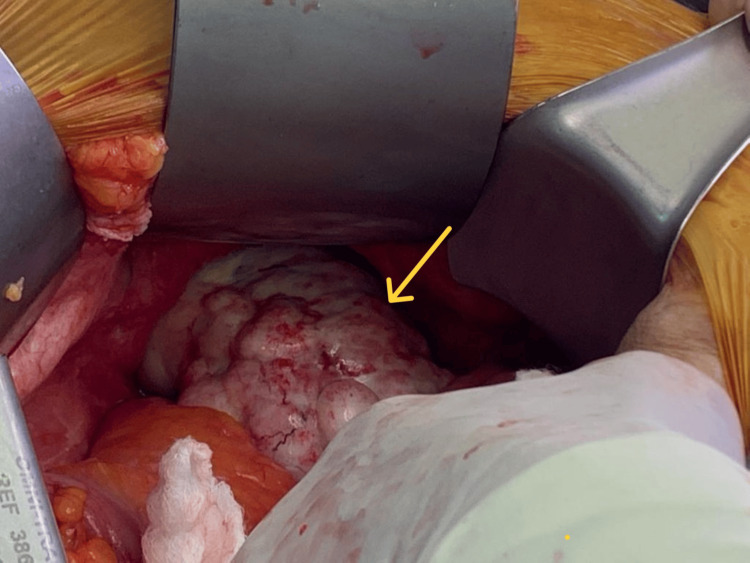
Pelvic deposit Intraoperative image showing a large pelvic deposit (yellow arrow) causing an intra-abdominal mass effect.

Due to suspected closed-loop obstruction, the patient underwent emergency exploratory laparotomy by midline laparotomy incision. Intraoperatively, two jejunal loops were found herniated through a 6 cm × 3 cm defect (Figure 6) in the transverse mesocolon. The herniated bowel loops appeared congested (Figure 7) but viable. A large pelvic mass (Figure 8) was noted adherent to the pelvic wall. Multiple metastatic deposits were seen on the liver surface, mesentery, and omentum.

The herniated bowel loops were reduced, and the mesocolic defect was closed using a non-absorbable suture. No bowel resection was required. The patient was started on an oral diet on the second postoperative day; her bowel was opened on the third postoperative day, and she was discharged in a stable condition on the fifth postoperative day.

## Discussion

Internal hernias are a rare but potentially life-threatening cause of bowel obstruction, with a high complication rate if they are not promptly diagnosed and treated. Internal hernias can be congenital or acquired. Transmesocolic hernias are among the rarest types of internal hernias, accounting for 5-10% of internal hernias and up to 0.6-5.8% of all small bowel obstructions [5]. These hernias occur when bowel loops herniate through a congenital or acquired defect in the transverse mesocolon. Predisposing factors include congenital mesenteric defects, surgical history, especially post-colectomy or gastric bypass, trauma or inflammation, and mass effect from intra-abdominal malignancies [6]. Acquired internal hernias are particularly associated with bariatric procedures, especially the Roux-en-Y gastric bypass, when the retrocolic Roux limb is created or mesenteric defects are not closed, which has caused an increase in the transmesocolic, transmesenteric, and retroanastomotic hernia [7,8].

In this case, the mesocolic defect was presumed to be acquired, possibly due to mechanical distortion from peritoneal carcinomatosis or by the pelvic mass. Alternatively, the defect may have been congenital and only became symptomatic due to recent tumor progression. Preoperatively, imaging was suggestive of small bowel obstruction but failed to identify the hernia or mesenteric defect, illustrating the diagnostic difficulty of internal hernias even with advanced imaging [9,10]. Prompt surgical exploration confirmed the diagnosis and allowed effective treatment before irreversible bowel ischemia occurred [11]. In recent years, laparoscopic surgery is becoming more feasible and can be a safe alternative for the repair of mesocolic hernias in suitable patients [12].

## Conclusions

Transmesocolic hernias are rare but must be considered in patients presenting with small bowel obstruction and no prior surgical history. Peritoneal malignancies may distort anatomical planes, can cause mass effect, and predispose to internal hernias. This case demonstrates that a high index of suspicion is required, especially in patients with metastatic intra-abdominal disease and non-specific imaging, and timely surgical intervention is critical to avoid bowel ischemia or infarction. Diagnostic laparoscopy or exploratory laparotomy remains the gold standard for both diagnosis and treatment when imaging is inconclusive.
